# Treatment of Refractory Centrofacial Melasma in a 64-Year-Old Female Using the NewMelan Depigmentation System: A Case Report

**DOI:** 10.7759/cureus.112676

**Published:** 2026-07-14

**Authors:** Maribel C Sanchez Meza

**Affiliations:** 1 Cirugia Plastica, Sculpt Clinic, Caracas, VEN

**Keywords:** case report, cosmetic dermatology, depigmentation therapy, facial hyperpigmentation, melasma, newmelan, refractory melasma, skin pigmentation, topical therapy, ultraviolet exposure

## Abstract

Melasma is a chronic acquired hyperpigmentation disorder that commonly affects sun-exposed areas of the face and may significantly impact quality of life. Treatment can be challenging because of frequent recurrence and variable responses to conventional therapies. This case report describes the management of refractory facial melasma in a 64-year-old patient with a history of prolonged ultraviolet exposure and persistent facial hyperpigmentation.

The patient previously underwent multiple treatment modalities, including chemical peels, mechanical peels, and laser-based therapies, without achieving satisfactory clinical improvement. Due to the limited response to these conventional approaches, treatment with the NewMelan Depigmentation System, a topical depigmenting formulation containing arbutin 1.5%, kojic acid 1%, and azelaic acid 1.5%, was initiated. The treatment protocol consisted of topical application three times daily during the first week, twice daily during the second week, and once daily during the subsequent two weeks, followed by maintenance therapy and continued photoprotection.

Progressive reduction in facial hyperpigmentation was observed throughout the treatment period. After one month of therapy, the pigmentation became markedly less noticeable, resulting in substantial cosmetic improvement and high patient satisfaction. Continued maintenance treatment and daily sun protection contributed to preservation of the clinical outcome.

This case highlights the potential role of the NewMelan Depigmentation System as a therapeutic option for patients with refractory facial melasma who have not responded adequately to conventional treatment modalities. Further clinical studies are warranted to evaluate long-term outcomes, safety, and efficacy in larger patient populations.

## Introduction

Melasma is a common acquired disorder of hyperpigmentation characterized by symmetrical brown to gray-brown macules and patches that primarily affect sun-exposed areas of the face [1]. The condition is more frequently observed in individuals with darker skin phototypes and is associated with multiple contributing factors, including ultraviolet radiation, genetic predisposition, hormonal influences, and environmental exposures [2,3]. Melasma is particularly prevalent in individuals with Fitzpatrick skin phototypes III to V, who are also more susceptible to treatment-related complications, including paradoxical post-inflammatory hyperpigmentation following chemical peels, laser therapies, and other procedural interventions [2,3]. Although melasma is a benign condition, its visible nature may significantly affect quality of life and psychosocial well-being [4].

Management of melasma remains challenging because of its chronic and recurrent nature [5]. Numerous treatment modalities have been proposed, including topical depigmenting agents, chemical peels, laser therapies, and combination treatment approaches [6,7]. However, treatment outcomes are often variable, and some patients fail to achieve satisfactory clinical improvement despite undergoing multiple therapeutic interventions [8,9].

Patients with refractory melasma represent a particularly difficult population to manage because persistent pigmentation may continue despite adherence to conventional treatment protocols [10]. As a result, alternative depigmentation strategies continue to be explored in an effort to improve clinical outcomes and patient satisfaction [11-15].

This report describes the clinical course of a 64-year-old patient with refractory facial melasma who previously underwent chemical peels, mechanical peels, and laser-based therapies without significant improvement. The patient subsequently received treatment with the NewMelan Depigmentation System, a topical depigmenting formulation containing arbutin 1.5%, kojic acid 1%, and azelaic acid 1.5%. The formulation was administered using a step-down regimen consisting of topical application three times daily during the first week, twice daily during the second week, and once daily during the subsequent two weeks, followed by once daily maintenance therapy in conjunction with strict photoprotection. This treatment protocol resulted in a substantial reduction of facial hyperpigmentation during follow-up.

## Case presentation

A 64-year-old female patient with Fitzpatrick skin phototype IV presented with refractory centrofacial melasma involving the bilateral malar regions and the forehead. The patient reported a history of significant ultraviolet exposure from outdoor activities and frequent beach visits. Hyperpigmentation had been present for several years and had progressively become a cosmetic concern.

Before presentation, the patient underwent multiple treatment modalities, including chemical peels of varying concentrations, mechanical peeling procedures, and laser-based therapies. Despite these interventions, little to no clinically significant improvement was observed, and the facial hyperpigmentation remained noticeable.

Given the refractory nature of the condition and the limited response to previous therapies, treatment with the NewMelan Depigmentation System was initiated. The topical depigmenting formulation contained arbutin 1.5%, kojic acid 1%, and azelaic acid 1.5%. The treatment protocol consisted of topical application three times daily during the first week, twice daily during the second week, and once daily during the third and fourth weeks. Following completion of the initial treatment phase, maintenance therapy and daily photoprotection were continued.

**Figure 1:**
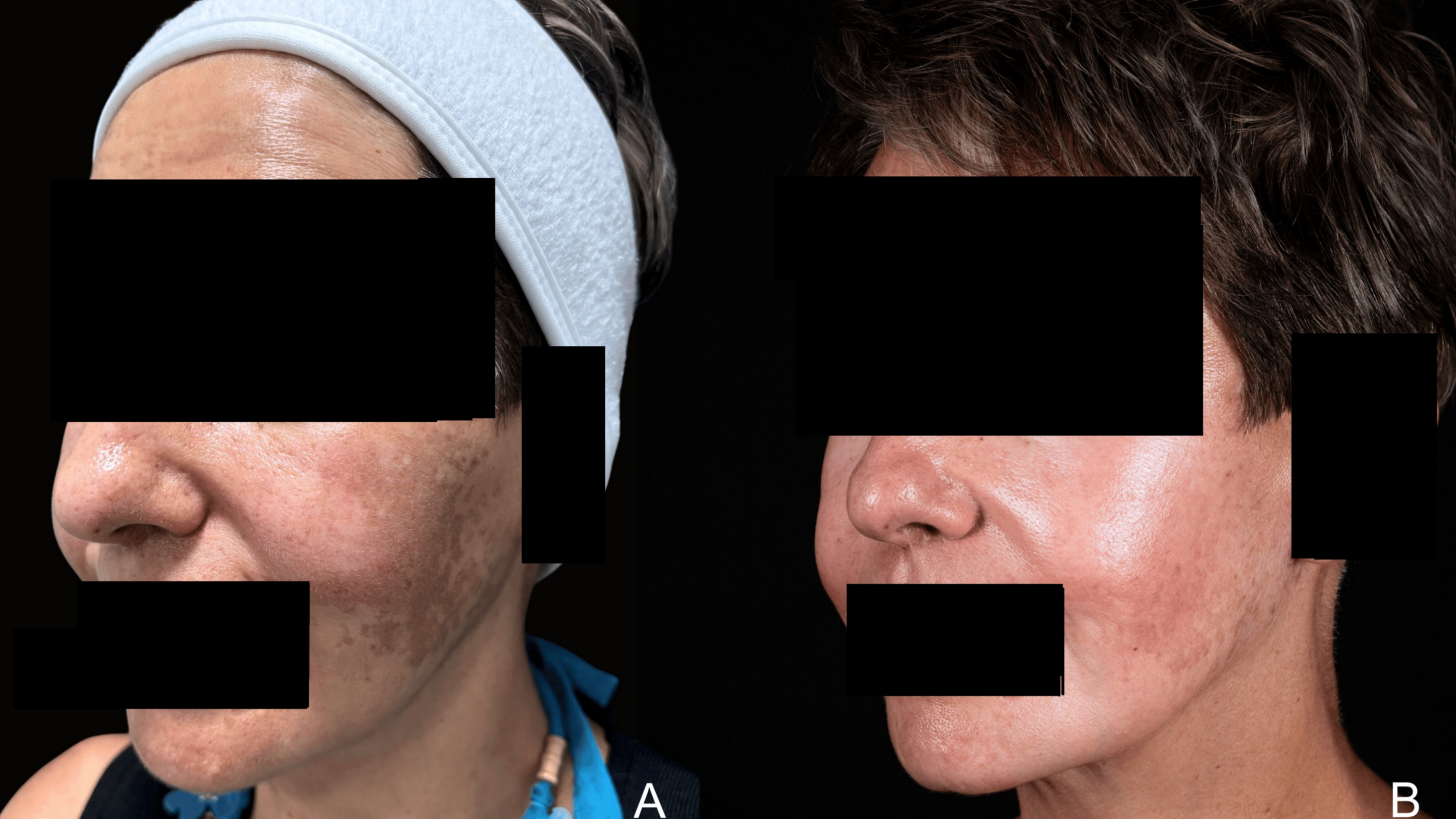
Clinical improvement of refractory facial melasma following treatment with the NewMelan Depigmentation System. (A) Baseline left oblique facial photograph demonstrating diffuse hyperpigmentation involving the malar and lateral facial regions prior to treatment. (B) Follow-up left oblique facial photograph demonstrating substantial reduction in pigmentation intensity after completion of the initial two-week NewMelan treatment protocol consisting of topical application three times daily during the first week and twice daily during the second week. Following the intensive treatment phase, NewMelan was continued as a long-term once-daily maintenance treatment.

**Figure 2:**
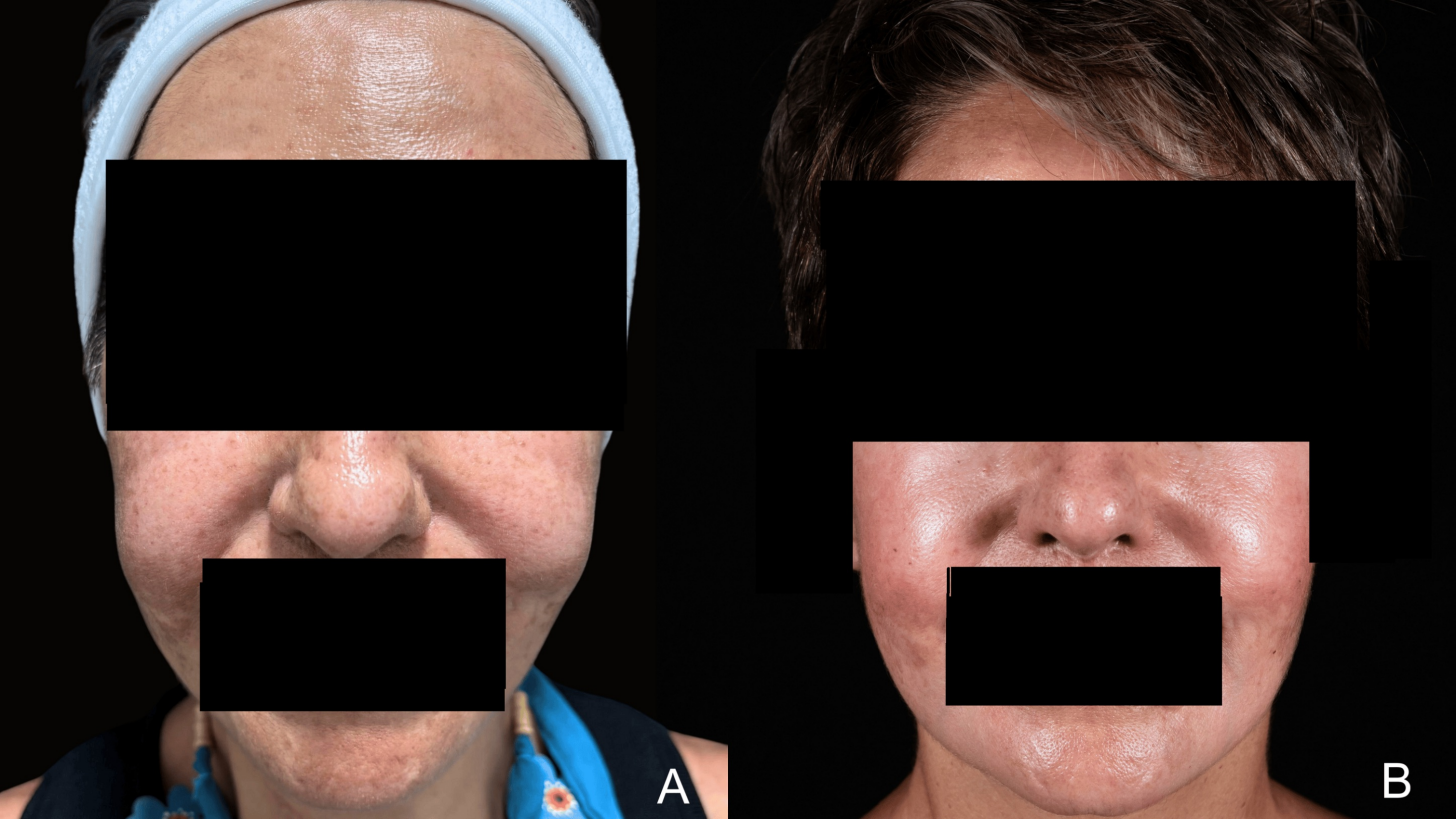
Frontal clinical photographs obtained before and after treatment with the NewMelan Depigmentation System. (A) Baseline frontal facial photograph demonstrating diffuse facial hyperpigmentation involving the bilateral malar regions and forehead prior to treatment. (B) Follow-up frontal facial photograph demonstrating substantial reduction in pigmentation intensity after completion of the initial two-week NewMelan treatment protocol consisting of topical application three times daily during the first week and twice daily during the second week. Following the intensive treatment phase, NewMelan was continued as a long-term once-daily maintenance treatment.

Baseline clinical photographs demonstrated symmetrical hyperpigmented patches distributed across the facial regions (Figure 1). Progressive reduction in pigmentation was observed throughout the treatment period. At the two-week follow-up, clinical examination revealed marked improvement in the appearance and intensity of the hyperpigmented lesions (Figure 2). The patient reported satisfaction with the cosmetic outcome and noted improvement in overall skin appearance.

**Figure 3:**
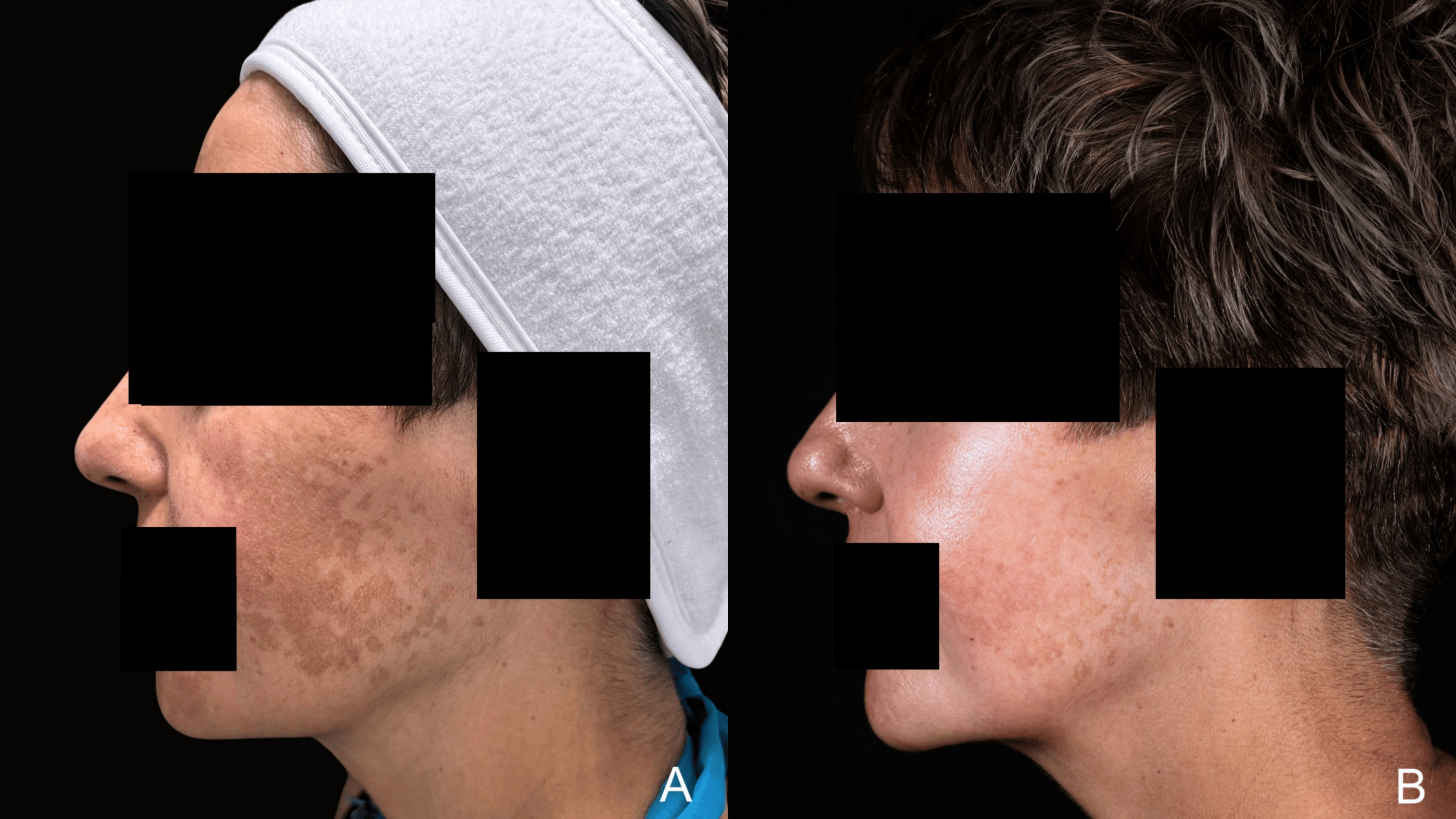
Lateral clinical photographs obtained before and after treatment with the NewMelan Depigmentation System. (A) Baseline lateral facial photograph demonstrating extensive hyperpigmentation involving the malar and lateral facial regions prior to treatment. (B) Follow-up lateral facial photograph demonstrating substantial reduction in pigmentation intensity after completion of the initial two-week NewMelan treatment protocol consisting of topical application three times daily during the first week and twice daily during the second week. Following the intensive treatment phase, NewMelan was continued as a long-term once-daily maintenance treatment.

Continued maintenance therapy and regular sunscreen use were recommended to minimize recurrence and preserve the clinical results. Follow-up photographs obtained after completion of the initial two-week treatment protocol demonstrated substantial reduction in visible facial pigmentation compared with baseline presentation (Figure 3).

## Discussion

Melasma remains one of the most challenging pigmentary disorders to manage because of its multifactorial pathogenesis and high tendency for recurrence [1]. Ultraviolet radiation, genetic susceptibility, hormonal influences, and environmental factors have all been implicated in its development [2,3]. Although numerous treatment options are available, including topical depigmenting agents, chemical peels, laser therapies, and combination approaches, treatment outcomes are often variable, and long-term maintenance is frequently required [4,5].

The patient described in this report had persistent facial melasma despite undergoing multiple conventional treatment modalities, including chemical peels, mechanical peeling procedures, and laser-based therapies. The limited response observed with these interventions highlights the difficulty of managing refractory cases and the need for alternative therapeutic approaches [6-8].

Following initiation of the NewMelan Depigmentation System, progressive reduction in facial hyperpigmentation was observed over the course of treatment. While the exact mechanisms responsible for the clinical improvement cannot be determined from a single case report, the observed outcome may be related to the combined effects of the formulation together with continued photoprotection measures [9,10]. Strict avoidance of excessive ultraviolet exposure and consistent sunscreen use likely contributed to the maintenance of clinical improvement [11].

This case also emphasizes the importance of patient adherence. Successful management of melasma often requires long-term commitment to treatment protocols and photoprotective strategies. Even when initial improvement is achieved, recurrence remains a recognized concern, particularly in individuals with continued exposure to triggering factors [12,13].

Several limitations should be considered when interpreting the findings of this report. First, this is a single-patient case report, and therefore, the results cannot be generalized to larger populations. Second, objective pigmentation measurements were not obtained, and assessment was based primarily on clinical observation and photographic documentation. Finally, longer follow-up is necessary to determine the durability of the observed improvement and the potential for recurrence.

Despite these limitations, the present case demonstrates substantial clinical improvement in a patient with refractory facial melasma following treatment with the NewMelan Depigmentation System.

Although the findings are encouraging, conclusions are limited by the nature of a single-patient case report. Further clinical studies with larger patient populations, objective pigmentation measurements, and longer follow-up periods are necessary to evaluate the safety, efficacy, recurrence rates, and long-term durability of this treatment approach [14,15].

## Conclusions

This case report describes substantial clinical improvement in a patient with refractory facial melasma following treatment with the NewMelan Depigmentation System after unsuccessful outcomes with chemical peels, mechanical peels, and laser-based therapies. Marked reduction in facial hyperpigmentation was observed during follow-up, resulting in improved cosmetic appearance and patient satisfaction. Clinical improvement was achieved following completion of the NewMelan step-down (3-2-1) treatment protocol and subsequent maintenance therapy. These findings suggest that the NewMelan Depigmentation System may represent a promising therapeutic option for selected patients with refractory melasma who have not responded adequately to conventional treatment modalities.
